# Osteoclasts Are Required for Hematopoietic Stem and Progenitor Cell Mobilization but Not for Stress Erythropoiesis in* Plasmodium chabaudi adami* Murine Malaria

**DOI:** 10.1155/2016/3909614

**Published:** 2016-01-21

**Authors:** Hugo Roméro, Christopher Warburton, Jaime Sanchez-Dardon, Tatiana Scorza

**Affiliations:** Département des Sciences Biologiques, Université du Québec à Montréal, Montréal, QC, Canada H3B 3H5

## Abstract

The anemia and inflammation concurrent with blood stage malaria trigger stress haematopoiesis and erythropoiesis. The activity of osteoclasts seems required for the mobilization of hematopoietic stem and progenitor cells (HSPC) from the bone marrow to the periphery. Knowing that BALB/c mice with acute* Plasmodium chabaudi adami* malaria have profound alterations in bone remodelling cells, we evaluated the extent to which osteoclasts influence their hematopoietic response to infection. For this, mice were treated with osteoclast inhibiting hormone calcitonin prior to parasite inoculation, and infection as well as hematological parameters was studied. In agreement with osteoclast-dependent HSPC mobilization, administration of calcitonin led to milder splenomegaly, reduced numbers of HSPC in the spleen, and their retention in the bone marrow. Although C-terminal telopeptide (CTX) levels, indicative of bone resorption, were lower in calcitonin-treated infected mice, they remained comparable in naive and control infected mice. Calcitonin-treated infected mice conveniently responded to anemia but generated less numbers of splenic macrophages and suffered from exacerbated infection; interestingly, calcitonin also decreased the number of macrophages generated* in vitro*. Globally, our results indicate that although osteoclast-dependent HSC mobilization from bone marrow to spleen is triggered in murine blood stage malaria, this activity is not essential for stress erythropoiesis.

## 1. Introduction

Quiescent hematopoietic stem cells (HSCs) reside in the bone marrow (BM) near the endosteum, in specific microenvironments called endosteal niches [1–3]. Low numbers of HSCs leave the BM and enter the blood stream in steady state conditions [4, 5], thereafter establishing in extramedullary sites of hematopoiesis like the spleen and liver, or returning to the BM [6, 7]. Recent studies involve bone resorbing osteoclasts in the homeostasis and mobilization of HSC and hematopoietic progenitor cells (HSPC) in conditions of stress, although this function remains controversial [8–11]. Indeed, stimulation of osteoclasts activity by Receptor Activator of Nuclear Factor Kappa-B Ligand (RANKL) increases HSPC mobilization through secretion of cathepsin K, which cleaves c-Kit-ligand and Stromal Cell-Derived Factor 1 (SDF-1), required for maintenance of endosteal niches. Phlebotomy or lipopolysaccharide (LPS) injection, two models of physiological stress, also increase osteoclastogenesis and trigger HSPC egression. Furthermore, the osteoclast inhibiting hormone calcitonin was reported to decrease HSPC mobilization in response to LPS injection [11]. However, contradictory evidences demonstrate that inhibition of osteoclast activity by bisphosphonate does not impair HSPC mobilization in response to Granulocyte-Colony Stimulating Factor (G-CSF) treatment [1, 10], suggesting that osteoclasts may only intervene in certain types of hematopoietic stresses.

Mobilization of HSPC is triggered by viral, bacterial, and* Plasmodium* infection [12–16], as well as by phenylhydrazine- (PHZ-) induced anemia in mice [17–19], but the contribution of osteoclasts in these responses has not been yet characterized. Blood stage malaria causes systemic inflammation and acute hemolytic anemia in mice [20], which are events that trigger stress hematopoiesis to generate phagocytic cells involved in parasite clearance and new erythrocytes to cope with anemia [21–24]. In murine malaria models, stress hematopoiesis associates with egression of HSPC from the BM to spleen, which becomes the major site of erythropoiesis [25, 26] and myelopoiesis [27, 28]. This mobilization is indirectly involved in the development of splenomegaly [16, 29] and extramedullary myelopoiesis [16], which are required in the resolution of acute parasitemia, through removal of parasitized red blood cells (pRBCs) by splenic macrophages [30–32].

We recently reported profound alterations in bone remodelling in mice with malarial or PHZ-induced hemolytic anemia [33], characterized by decreased bone formation, reduced osteoclastogenesis, and balance resulting in bone mass loss. Knowing these alterations, the contribution of osteoclasts in HSPC egression, stress erythropoiesis, and resolution of* Plasmodium chabaudi adami* DK infection was investigated in calcitonin-treated mice. Calcitonin is a peptide hormone known to inhibit osteoclasts bone resorption and osteoclast differentiation [34–36]. Our data indicate that calcitonin causes a significant drop in the basal activity of osteoclasts and partially interferes with the egression of HSPC from BM to spleen in blood stage malaria. However, the development of splenic stress erythropoiesis is not affected by this treatment. Unexpectedly, treatment with calcitonin exacerbates parasitemia* in vivo* and interferes with macrophage differentiation and proliferation* in vivo *and* in vitro*.

## 2. Materials and Methods

All procedures in mice were approved by the Animal Care Committee of the Université du Québec à Montréal (protocol 0210-677-0211) and according to relevant national and international guidelines.

### 2.1. Mice and* In Vivo* Treatments

Female BALB/c mice (Charles River, Canada) aged 4–6 weeks were used in all experiments. Mice were maintained in temperature controlled conditions (22 ± 1°C) under a 12-hour light cycle (7:00 to 19:00), having free access to osmosed water and rodent chow 5075 (Charles River, Canada). Mice were euthanized by isoflurane inhalation followed by CO_2_. Mice were infected with the* P. c. adami* DK nonlethal strain, originally isolated from* Thamnomys rutilans* and kindly provided by Dr. David Walliker, University of Edinburgh (Congo-Brazzaville, 1972) [37].

To evaluate the contribution of osteoclasts in HSPC mobilization during* Plasmodium* infection, mice received 200 *μ*L salmon calcitonin (5 *μ*g/mL in PBS) (Biotrend, Switzerland) during 5 consecutive days by the intraperitoneal route and were infected with 10^5^ *P. c. adami *DK pRBC in PBS by the intravenous route on the 3rd day of treatment. Mice were euthanized on days 5, 8, and 10 after infection for analysis of hematopoietic parameters and cytokines in plasma by ELISA (BioLegend, USA). To assess the effect of calcitonin-dependent osteoclasts inhibition on the erythropoietic responses to experimental hemolytic and nonhemolytic anemia, mice were injected intraperitoneally with 200 *μ*L of salmon calcitonin (5 *μ*g/mL in PBS) (Biotrend, Switzerland) during 5 consecutive days. Hemolytic anemia was induced in mice by peritoneal administration of PHZ (Sigma Aldrich, Canada) at 40 mg/Kg of body weight in 200 *μ*L of PBS, on the 3rd and 4th days of calcitonin treatment. To induce nonhemolytic anemia, the submandibular vein was incised with a 4 mm lancet (Medipoint, USA) and 300 *μ*L of blood was withdrawn on the 3rd day of calcitonin treatment. Mice received an equal volume of physiologic saline solution by the intraperitoneal route immediately after phlebotomy. In certain experiments with infected mice, 100 *μ*L DiD-loaded anionic liposomes (Formumax, USA) were administered intravenously one day prior to calcitonin treatment.

### 2.2. Determination of Hemoglobin, Reticulocytes, and Parasitemia in Blood

Hemoglobin levels were monitored daily by diluting 2 *μ*L tail-vein blood in 500 *μ*L Drabkin's solution (Sigma Aldrich, Canada). Following 15 min of incubation in the dark, 200 *μ*L of blood was transferred into 96-well plates (Costar, USA) in duplicate and absorbance was measured at 540 nm in a microplate reader. Values were converted to g/dL using a standard curve of rat hemoglobin (Sigma Aldrich, Canada) prepared in Drabkin's solution. All samples were assessed in duplicate. To estimate the percentages of reticulocytes in blood, 1 *μ*L of tail-vein blood was collected in 1 mL of PBS. The cell suspensions were then stained with anti-CD71-FITC antibody (BioLegend, USA) and incubated at 4°C for 30 min. Data were acquired with an Accuri C6 (Becton Dickinson, USA) and analyzed with the FlowJo software (Tree Star). In parallel, parasitemia was measured daily in methanol fixed blood smears stained with a 10% Giemsa solution in PBS during 15 minutes.

### 2.3. Cell Isolation and Flow Cytometry

Bone marrow single-cell suspensions were prepared by removal of tibia and femur epiphyses and flushing the marrow into 100 *μ*L of PBS by centrifugation of 300 g during 5 minutes. Spleen single-cell suspensions were obtained by mechanically dissociating the spleen in a 60 mm petri dish containing 5 mL of PBS. Recovered cells from the BM and the spleen were counted and resuspended at 10^7^ cells/mL in RPMI 1640, supplemented with 10%, penicillin (100 U/mL), and streptomycin (100 mg/mL) (Invitrogen, USA). Cells were distributed at 100 *μ*L/tube into 12 × 75 polypropylene tubes (Sarstedt, Canada). For assessment of erythroid populations, cells were stained with 1 *μ*g of TER119-PE and anti-CD71-FITC (clone RI7217) antibodies. For additional analysis, RBCs in spleen cell suspensions were lysed with the RBC lysing buffer Hybri-Max (Sigma Aldrich, Canada). HSCs were stained with 10 *μ*L of the biotinylated lineage antibody cocktail (CD3, Ly6/6C, B220, CD11b, and Ter119) and 1 *μ*g of streptavidin-APC, anti-Sca1-FITC (clone D7), and anti-CD117-PE (clone 28B). Mature macrophages were stained with 1 *μ*g of anti-CD11b-FITC (clone M1/70) and anti-F4/80-PE (clone BM8). Dead cells were excluded from analysis with 7-amino-actinomycin D (7-AAD) (BioLegend, USA) staining. All antibodies were from BioLegend, USA. Analytical flow cytometry was performed on an Accuri C6 (Becton Dickinson, USA) and data was analyzed using FloJo software (Tree Star).

### 2.4. Quantification of Macrophage Colony Stimulating Factor and C-Terminal Telopeptide by ELISA

ELISA for quantification of macrophage colony-stimulating factor (M-CSF, Promokine, Canada) and C-terminal telopeptide (CTX, Mybiosource, USA) were performed following the manufacturer's instructions. Briefly, 96-well ELISA MaxiSorp plates (Nunc, USA) were coated overnight with 60 *μ*L of capture antibody at 4°C. Wells were washed 4 times with washing buffer (0, 05% Tween 20 in PBS, 300 *μ*L) and incubated with blocking buffer (1% BSA in PBS) for 1 hour. Following 4 washes, 60 *μ*L of samples and standards was added and incubated for 2 hours at room temperature, the plates were washed, and 60 *μ*L of detection antibody was added and incubated for 2 hours at room temperature. After 4 washes, 60 *μ*L of avidin-HRP was added and incubated for 30 min at room temperature, after which wells were washed 4 times. Sixty *μ*L of tetramethylbenzidine (TMB) substrate solution (Sigma-Aldrich, Canada) was added and incubated for 20 min at room temperature in the dark, and the reaction was stopped by adding 60 *μ*L 1 M H_2_SO_4_. The absorbance was determined with a microplate reader set at 450 nm.

### 2.5. Colony-Forming Unit Assays

Single-cell suspensions from the spleen and BM were prepared at 1 × 10^6^ and 2 × 10^5^ cells/mL, respectively, in RPMI 1640, supplemented with 10%, penicillin (100 U/mL), and streptomycin (100 mg/mL) (Invitrogen, USA) and 0.3 mL was added into 3 mL of MethoCult 03434 (StemCell Technologies, USA). The cell suspension was vortexed, allowed to stand for 10 min, and dispensed into 35 mm culture dishes (StemCell Technologies, USA) using a 16-gauge blunt-end needle and a 3 mL syringe, 1.1 mL per dish in duplicate. The cultures were incubated at 37°C, 5% CO_2_ in air, and 95% humidity for 8 days after which Colony-Forming Unit Macrophages (CFU-M) were identified and counted.

### 2.6. Macrophages Differentiation* In Vitro*

Single-cell suspensions from BM were prepared at 1 × 10^6^ cells/mL in RPMI 1640 supplemented with 10% FBS, penicillin (100 U/mL) (Sigma, USA), streptomycin (100 mg/mL) (Sigma, USA), 30% L929 cells supernatant (ATCC, USA), and 0, 125, 250, and 500 ng/mL of salmon calcitonin (Biotrend, Switzerland). After 6 days of incubation at 37°C, 5% CO_2_ in air, and 95% humidity, cells were harvested and macrophage differentiation was assessed by flow cytometry with CD11b-FITC (BioLegend, USA) and F4/80-PE (BioLegend, USA) staining. To evaluate cell proliferation, a 3-(4,5-dimethylthiazol-2-yl)-5-(3-carboxymethoxyphenyl)-2-(4-sulfophenyl)-2H-tetrazolium inner salt/phenazine methosulfate (MTS/PMS) solution (Promega, USA) was added to cell culture in a ration 2 : 10. After 2 h incubation at 37°C, 5% CO_2_ in air, and 95% humidity, absorbance was measured at 490 nm in a microplate reader.

### 2.7. Statistical Analysis

Statistical analysis was performed with a one-way ANOVA analysis of variance followed by Tukey's posttest for comparison of more than two groups and an unpaired *t*-test for comparison of two groups. ^*∗*^ *P* < 0.05; ^*∗∗*^ *P* < 0.01; ^*∗∗∗*^ *P* < 0.001 represent comparisons in respect to respective controls.

## 3. Results 

### 3.1. Administration of Calcitonin Enhances Parasitemia but Does Not Affect Splenic Stress Erythropoiesis

**Figure 1:**
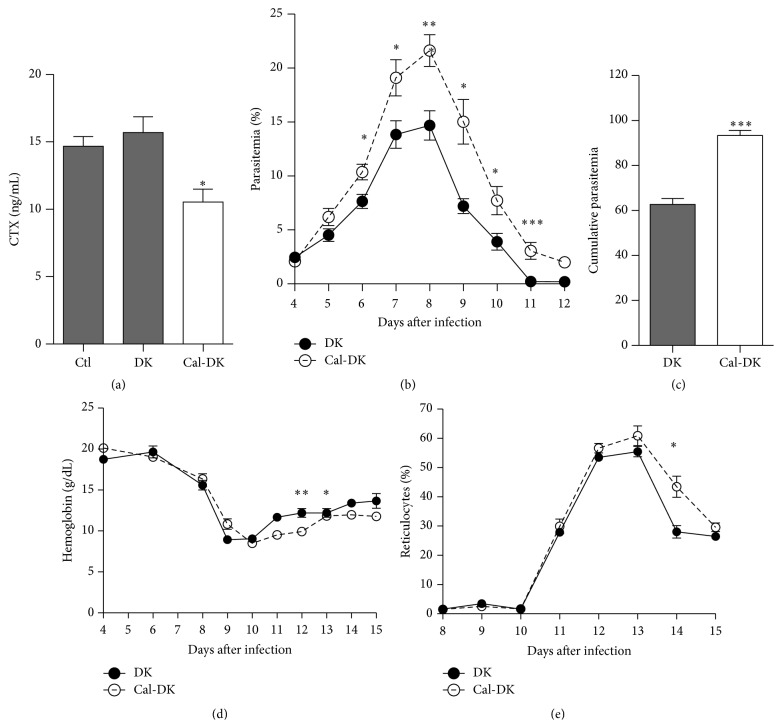
Impact of calcitonin on* Plasmodium chabaudi adami* parasitemia and anemia. Mice were treated with calcitonin (Cal-DK) or PBS (Ctl and DK) during 5 consecutive days.* P. c. adami* DK parasites (10^5^ pRBCs) were inoculated on the 3rd day of treatment. Four mice of each infected group and 4 uninfected controls were sacrificed at 5 day after infection and C-telopeptide of type I collagen (CTX) levels were assessed in the plasma by ELISA (a). Parasitemia was monitored daily for determination of the kinetics of infection (b) and cumulative parasitemia (c). Hemoglobin levels (d) and reticulocytosis (e) were measured in the blood. Data are mean ± SEM and represent the compilation of three experiments (a total of 14–18 mice per infected group); values are compared using a nonparametric Student's *t*-test. ^*∗*^ *P* < 0.05; ^*∗∗*^ *P* < 0.01; ^*∗∗∗*^ *P* < 0.001.

In order to investigate the contribution for osteoclasts in malarial stress hematopoiesis with the* P. c. adami* DK infection model, BALB/c mice were treated with calcitonin two days prior to infection and three days following infection. Resolution of infection and subsequent stress erythropoiesis were monitored and compared in control and calcitonin-treated mice. For comparative purposes, the calcitonin dose and administration scheduled reported by Kollet et al. were used [11]. As expected, significantly lower plasma concentrations of CTX were measured in calcitonin-treated mice on day 5 after infection, confirming inhibition of osteoclast activity (Figure 1(a); *P* < 0.05). Parasitemia was exacerbated in calcitonin-treated mice throughout patent infection (Figure 1(b); *P* < 0.01), resulting in higher cumulative parasite burden (Figure 1(c); *P* < 0.001). Consequent to higher parasitemia, hemoglobin levels were relatively lower in calcitonin-treated mice on days 11 and 12 after infection (Figure 1(d); *P* < 0.01 and *P* < 0.05, resp.). Concurrent with enhanced anemia, the percentages of reticulocytes (CD71^+^ cells) were higher in calcitonin-treated mice on day 14 after infection (Figure 1(e)).

**Figure 2:**
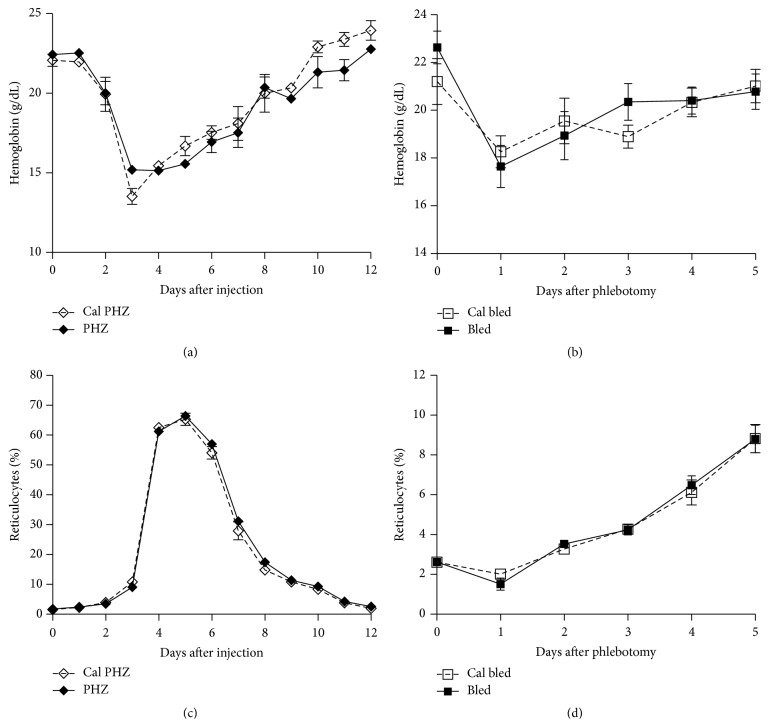
Effect of calcitonin on postexperimental anemia recovery. Mice were treated with calcitonin (Cal-Bled and Cal-PHZ) or PBS (Bled and PHZ) during 5 consecutive days. On the 3rd day of treatment, mice were phlebotomized (Bled and Cal-Bled; *n* = 11) or received phenylhydrazine injections (PHZ and Cal-PHZ; *n* = 3) and blood hemoglobin concentration (a and b) and reticulocytosis (c and d) were monitored daily. Data are mean ± SEM and values are compared using a nonparametric Student's *t*-test.

The erythropoietic responses to anemia were also followed in PHZ-treated or phlebotomized mice (Figures 2(a) and 2(b)). In accordance with the responses to infection, comparable reticulocytosis occurred in control and calcitonin-treated mice under these two experimental conditions (Figures 2(c) and 2(d)).

### 3.2. Administration of Calcitonin Promotes Retention of HSC in the Bone Marrow and Decreases Malarial Splenomegaly

**Figure 3:**
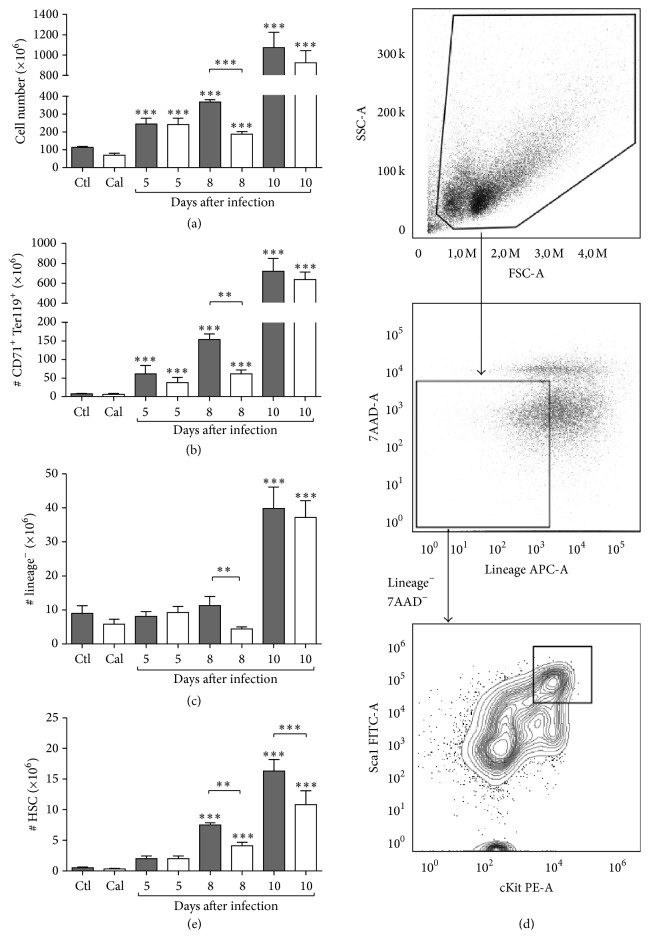
Modulation of splenic hematopoiesis during* Plasmodium* infection by calcitonin. Mice were treated with calcitonin (white bars) or with PBS (grey bars) during 5 consecutive days and were infected with* P. c. adami DK parasites* (10^5^ pRBCs) on the 3rd day of treatment. Data from uninfected controls (Ctl, Cal *n* = 3) is included for comparison. Infected mice were sacrificed at 5 (*n* = 8), 8 (*n* = 8–12), and 10 (*n* = 3-4) days after infection and the spleen was aseptically recovered for analysis of numbers of total cells (a), erythroid cells (b), lineage^−^ cells (c), and hematopoietic stem cells (e) by cytofluorometry. Identification panel of hematopoietic stem cells (Lineage-7AAD^−^Sca1^+^cKit^+^) by cytofluorometry is also represented (d). Data are mean ± SEM and values are compared to respective uninfected control mice and between the two infected groups using a one-way ANOVA test. ^*∗*^ *P* < 0.05; ^*∗∗*^ *P* < 0.01; ^*∗∗∗*^ *P* < 0.001.

Considering the crucial role of splenic stress hematopoiesis in the control of* Plasmodium* infection [16, 28] and anemia [21, 25, 26, 38], the effect of calcitonin on splenic hematopoietic parameters was investigated early (day 5 after infection), at peak parasitemia (day 8), and following resolution of peak infection (day 10). As expected, spleen cellularity increased in mice with malaria (Figure 3(a)), and this effect was apparent and comparable in control and calcitonin-treated mice until day 5 after infection (Figure 3(a)). However, reduced numbers of splenocytes (Figure 3(a); *P* < 0.001) and reduced splenic index (Supplementary Figure 1 in Supplementary Material available online at http://dx.doi.org/10.1155/2016/3909614; *P* < 0.01) were measured in calcitonin-treated mice at peak infection, indicating milder splenomegaly.

Parallel monitoring of the erythroid populations confirmed higher numbers of splenic CD71^+^ Ter119^+^ cells in infected mice from day 5 after infection, which increased thereafter (Figure 3(b); *P* < 0.001); this response was significantly attenuated in calcitonin-treated mice at the time of peak infection (Figure 3(b); *P* < 0.01). In respect to splenic lineage negative cells, their numbers increased significantly compared to uninfected controls only after peak infection. Major differences were only apparent on day 8 after infection, reflected by significantly lower lineage negative cell numbers in calcitonin-treated infected mice (Figure 3(c); *P* < 0.01). In contrast, administration of calcitonin significantly reduced the number of splenic lineage negative cKit^+^ Sca1^+^ cells (LSK) at peak infection, as well as 10 days after infection (Figure 3(e); *P* < 0.01, 0.001, resp.).

The analysis of lymphoid populations in spleen revealed increased numbers of CD90^+^ cells (Supplementary Figure 2(A); *P* < 0.001) and CD19^+^ cells (Supplementary Figure 2(B); *P* < 0.001) at peak infection; treatment with calcitonin resulted in lower CD19^+^ cell numbers (Supplementary Figure 2(B); *P* < 0.05). In respect to splenic myeloid cells, calcitonin did not affect the characteristic rise in CD11b^+^ cells (Supplementary Figure 2(C); *P* < 0.001) and FcERIa^+^ cKit^+^ cells at peak infection (Supplementary Figure 2(D); *P* < 0.01).

**Figure 4:**
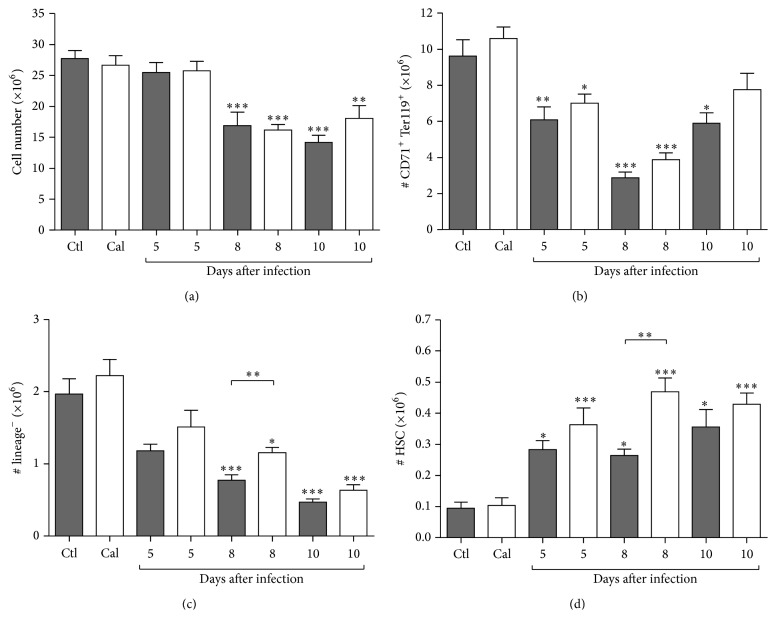
Impact of calcitonin on bone marrow hematopoiesis during* Plasmodium* infection. Mice were treated with calcitonin (white bars; Cal) or with PBS (grey bars; Ctl) during 5 consecutive days and were infected with* P. c. adami DK parasites* (10^5^ pRBCs) on the 3rd day of treatment. Data from uninfected controls (Ctl, Cal *n* = 3) is included for comparison. Mice were sacrificed 5 (*n* = 8), 8 (*n* = 8–12), or 10 (*n* = 3-4) days after infection, and femoral and tibia bone marrow was aseptically recovered for analysis of numbers of total cells (a), erythroid cells (b), lineage^−^ cells (c), and hematopoietic stem cells (d) by cytofluorometry. Data are mean ± SEM from 3 independent experiments (a total of 19–24 mice per infected group); values are compared to respective uninfected control mice and between the two infected groups using a one-way ANOVA test. ^*∗*^ *P* < 0.05; ^*∗∗*^ *P* < 0.01; ^*∗∗∗*^ *P* < 0.001.

Analysis of BM hematopoietic parameters confirmed the drops previously reported in BM cellularity of infected mice [39] (Figure 4(a)), and this effect was not modified by calcitonin. Evaluation of the erythroid populations in the BM showed similar drops in CD71^+^ Ter119^+^ cell numbers in the two groups of infected mice (Figure 4(b)). Compared to uninfected mice, the number of BM lineage negative cells significantly dropped on days 8 and 10 after infection in infected mice (Figure 4(c)), becoming relatively higher in calcitonin-treated mice when compared to infected controls at peak infection (Figure 4(c); *P* < 0.01). The numbers of LSK cells also increased in the BM of infected mice and were significantly higher in calcitonin-treated mice on day 8 after infection (Figure 4(d), *P* < 0.01).

### 3.3. Calcitonin Decreased Macrophages Number in Spleen and Altered Their Production during* Plasmodium* Infection

**Figure 5:**
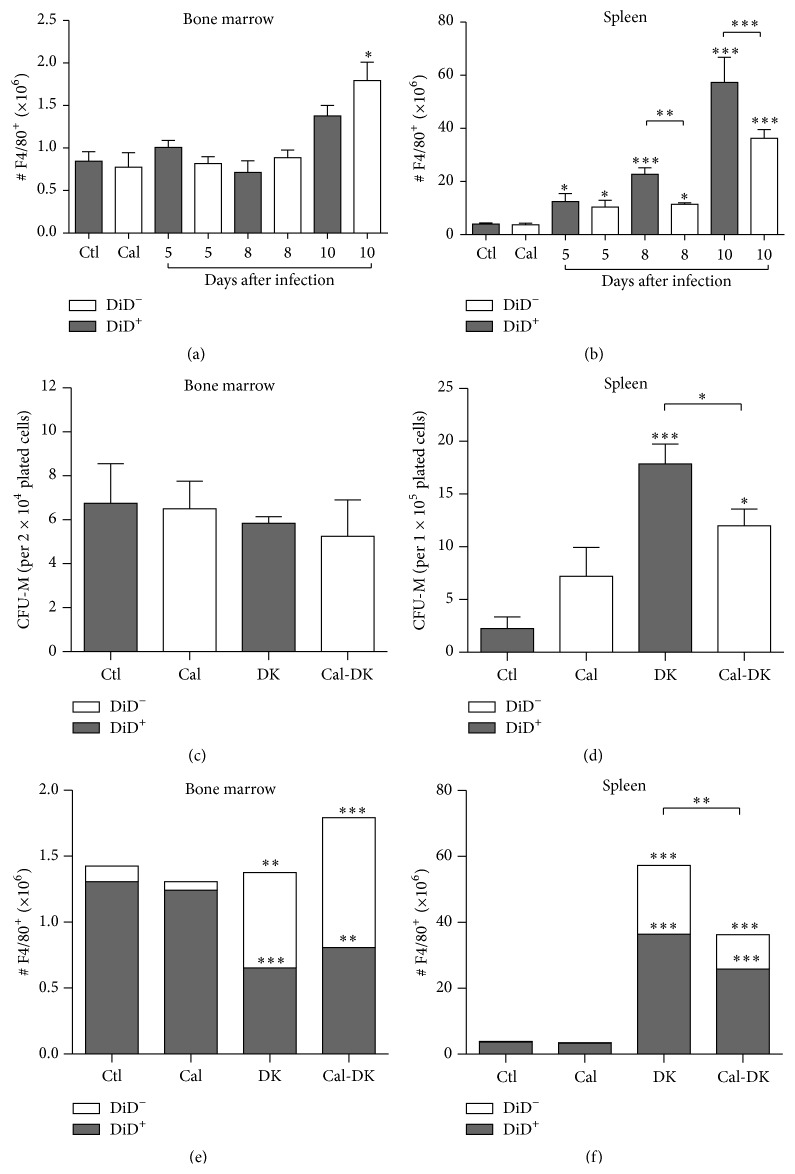
Modulation of macrophages population by calcitonin during* Plasmodium* infection. Before experimentation, mice were injected intravenously with DiD-liposomes for staining of phagocytic cells. Mice were treated with calcitonin (white bars; Cal) or with PBS (grey bars; Ctl) during 5 consecutive days and were infected with* P. c. adami DK parasites* (10^5^ pRBCs) on the 3rd day of treatment. Data from uninfected controls (Ctl, Cal *n* = 3) is included for comparison. Mice were sacrificed at 5 (*n* = 8), 8 (*n* = 8–12), and 10 (*n* = 3-4) days after infection and macrophages (F4/80^+^ cells) were assessed by cytofluorometry in bone marrow (a) and spleen (b). At 8 days after infection, the numbers of Colony Forming Unit-Macrophage (CFU-M) were determined in 12-day bone marrow (c) and spleen (d) cultures using MethoCult 03434 media. At 10 days after infection, the numbers of DiD^+^ and DiD^−^ negative macrophages (F4/80^+^ cells) were evaluated by cytofluorometry in bone marrow (e) and spleen (f). Data are mean ± SEM from 3 independent experiments (with a total of 19–24 mice per infected group); values are compared to respective uninfected control mice and between the two infected mice groups using a one-way ANOVA test. ^*∗*^ *P* < 0.05; ^*∗∗*^ *P* < 0.01; ^*∗∗∗*^ *P* < 0.001.

As reviewed by Chua et al. [24], phagocytic macrophages are essential for clearance of pRBCs, and dysregulation of their number or function negatively affects resolution of* Plasmodium* infection. To determinate whether the increased parasitemia observed in calcitonin-treated mice was due to restricted production of macrophages, the numbers of F4-80^+^ cells were analyzed in the BM and spleen. Relative to uninfected mice, the infection did not provoke major effects on BM F4/80^+^ cells, but their numbers were significantly higher in calcitonin-treated mice on day 10 after infection (Figure 5(a); *P* < 0.05). In contrast to the BM, the infection caused an important increase in splenic F4/80^+^ cells, and this effect was significantly attenuated in calcitonin-treated mice on days 8 and 10 after infection (Figure 5(b); *P* < 0.01, 0.001, resp.). Analysis of progenitors Colony Forming Unit-Macrophage (CFU-M) did not reveal major modifications in the BM of infected mice (Figure 5(c)), but lower numbers of CFU-M generated from the spleen of calcitonin-treated mice on day 8 after infection (Figure 5(d); *P* < 0.05). Interestingly, serum M-CSF levels remained comparable in infected and uninfected mice (Supplementary Figure 3).

Altogether, our results suggest altered production of macrophages in calcitonin-treated and infected mice. To discriminate the impact of calcitonin on preexisting versus* de novo* generated macrophages, DiD anionic liposomes were administered prior to infection, to specifically stain all phagocytic cells including macrophages. DiD (DiIC18_5_) is a viable lipophilic fluorescent dye weakly fluorescent in water, but highly fluorescent and photostable when incorporated into cell membranes [40], and allows discriminating mature macrophages (DiD positive) from* de novo* generated macrophages (DiD negative) (Supplementary Figure 4). The lipid composition of the anionic liposomes was originally designed to efficiently deplete phagocytic cells* in vivo* and* in vitro* when loaded with clodronate, and as nonphagocytic cells are not affected, this tool is efficient for targeting of macrophages [41, 42]. Compared to uninfected controls, in which the majority of BM macrophages were DiD^+^, approximately 50% F4/80^+^ DiD^+^ cells were found in infected mice on day 10 after infection, suggesting mobilization of mature macrophages from the BM and production of new (DiD^−^) macrophages (Figure 5(e); *P* < 0.01). The numbers of splenic F4/80^+^ DiD^+^ cells drastically increased on day 10 after infection, indicating recruitment of mature macrophages to the spleen after peak infection (Figure 5(f); *P* < 0.001). F4/80^+^ DiD^−^ cells also increased during infection, but this response was half-fold in calcitonin-treated mice (Figure 5(f); *P* < 0.01), suggesting deficient* de novo* production of macrophages.

### 3.4. Calcitonin Inhibits* In Vitro* Differentiation of Macrophages

**Figure 6:**
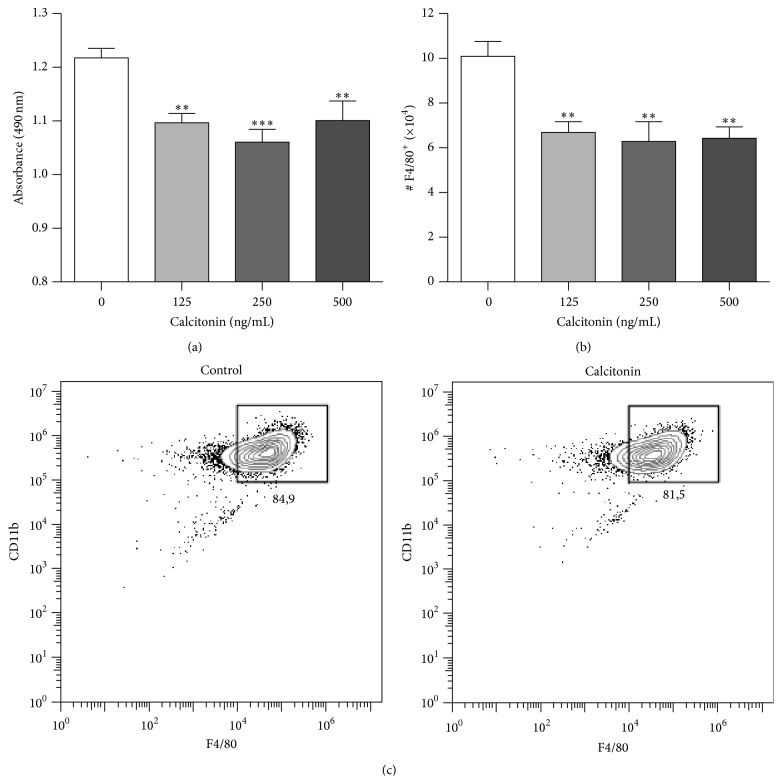
Impact of calcitonin on macrophage differentiation* in vitro*. Nonadherent cells of bone marrow at 10^6^ cells/mL were cultured during 5 days in macrophage differentiation medium with different concentrations of calcitonin (*n* = 7–9). Cell proliferation was evaluated by MTS/PMS assay (a) and macrophage counts (F4/80^+^ cells) were assessed by cytometry following identification panel (c). Data are mean ± SEM and values are compared to controls using a one-way ANOVA test. ^*∗*^ *P* < 0.05; ^*∗∗*^ *P* < 0.01; ^*∗∗∗*^ *P* < 0.001.

Calcitonin was originally documented to inhibit osteoclasts bone resorption activity and osteoclast differentiation [34–36]. In order to investigate whether the lower macrophage numbers found in calcitonin-treated mice are concurrent with direct action of this hormone on myeloid precursors, the impact of calcitonin on differentiation of macrophage progenitors was evaluated* in vitro*. Our data indicates reduced proliferation (Figure 6(a); *P* < 0.01) and lower numbers of of F4/80^+^ cells in BM cultures stimulated with M-CSF (Figure 6(b); *P* < 0.01).

## 4. Discussion

Herein, we evaluated the relative contribution for osteoclasts in the egression of HSPC from the BM to the spleen during blood stage malaria, using the well-characterized* P. c. adami* DK infection model in BALB/c mice. We previously compared bone remodelling markers in mice suffering from acute hemolytic anemia caused by* P. c. adami* infection or PHZ injection. These conditions are characterized by reduced bone mineralization and bone formation, as well as reduced numbers of osteoclasts and osteoclasts progenitors in the BM [33]. Considering that the levels of CTX, indicative of osteoclast-dependent bone resorption, remained comparable in infected, PHZ-treated, and naive mice [33], we concluded that the decreased bone mass density found in mice with hemolytic anemia was concurrent with imbalance favoring bone resorption, as has been reported for other hemolytic conditions [43, 44]. Herein, calcitonin was administered to mice three days prior to and during the first two days of infection to block bone resorption. Interestingly, plasma CTX levels were comparable in naive and infected mice on day 5 after infection, suggesting no major stimulation of bone resorption in this murine malaria model. The fact that CTX levels were lower in calcitonin-treated mice confirmed an inhibitory action of this peptide hormone on steady state osteoclast activity.

In our experimental malaria model, the number of HSPC increased in a comparable manner in the BM of calcitonin-treated and control-infected mice on day 5 after infection, with no major effects in the spleen. However, at peak infection, calcitonin-treated mice had higher numbers of HSPC in the BM and lower numbers of HSPC in the spleen, also developing relatively milder splenomegaly. These results suggest that osteoclast-dependent mobilization of HSPC to the spleen is partially responsible for splenomegaly in murine malaria. Comparable recruitment of HPSC to the spleen has been reported in C57BL/6 mice infected with* P. chabaudi* AS. In this malaria model, IFN-*γ* signalling and concurrent secretion of C-C motif ligand 2/7 (CCL2/CCL7) chemokines by BM stromal cells seem required for HSPC mobilization [16]. Interestingly, IFN-*γ* signalling indirectly stimulates the activity of osteoclasts [45], which may stimulate their effects in HSPC mobilization.

In opposition to reduced HSPC mobilization, calcitonin did not affect splenic stress erythropoiesis. Appearance of circulating reticulocytes is considered an ultimate marker for functional erythropoiesis (as reviewed in [46]), and our data indicate comparable reticulocytosis in calcitonin-treated and control mice in response to malarial anemia, despite reduced mobilization of HSPC and reduced numbers of erythroid progenitors in the spleen. Accordingly, calcitonin did not affect reticulocytosis in mice recovering from anemia caused by bleeding or PHZ-treatment.

Calcitonin-treated mice developed higher parasitemia throughout the period of patent infection, resulting in enhanced anemia on days 11 and 12 after infection. In logic accordance with an exacerbated anemia, higher reticulocytosis occurred in these mice on day 14 after infection, which suggested that the drop in splenic erythroid progenitors on day 8 after infection did not have major effects on the recovery from anemia. In this context, stress Burst Forming Unit-Erythroid (BFU-E) with self-renewal properties have been reported, which efficiently generate cKit^+^ CD71^+^ Ter119^+^ cells in response to erythropoietin (EPO), bone morphogenetic protein-4 (BMP-4), stem cell factor (SCF), and hypoxia [47, 48]. As comparable numbers of cKit^+^ CD71^+^ Ter119^+^ cells were measured in control and calcitonin-treated mice on day 8 after infection (data not shown), we conclude that osteoclast-dependent HSPC mobilization from the BM is not essential for splenic stress erythropoiesis in murine blood stage malaria.

Analysis of lymphocytic and granulocytic cell populations in the spleen at peak infection revealed no modifications in the numbers of T cells, mast cells, and myeloid cells in calcitonin-treated mice, although decreased numbers of B cells were noticed. The exacerbated parasitemia measured in calcitonin-treated mice may be concurrent with compromised parasite killing by macrophages. Indeed, an important proportion of pRBCs circulate in the blood stream [49] and their elimination by spleen red pulp macrophages is pivotal for the control of infection [50–52]. Calcitonin-treated mice, which developed exacerbated parasitemia, had fewer numbers of macrophages in the spleen on days 8 and 10 after infection and, accordingly, reduced numbers of CFU-M generated from the spleen of these mice. B cells only intervene in the late resolution of blood stage malaria [53], and the reduction caused by calcitonin on CD19^+^ cells numbers is not expected to affect the resolution of* P. c. adami* infection. In contrast, C-C chemokine receptor type 2- (CCR2-) dependent migration of monocytes from the BM to spleen [54] and splenic myelopoiesis are essential for the control of* P. chabaudi* infection [16]. Administration of DiD-loaded liposomes prior to calcitonin treatment and infection efficiently labelled macrophages, allowing following their fate and distinguishing newly generated macrophages* in vivo*. In respect to noninfected controls, we evidenced comparable drops in the number of DiD^+^ macrophages in the BM and their concurrent increase in the spleen during infection. These results suggest that calcitonin does not alter the mobilization of monocytes/macrophages from the BM nor their recruitment to spleen. However, the fact that the numbers of splenic DiD^−^ macrophages were significantly lower in calcitonin-treated mice on day 10 after infection rather indicates impaired* de novo* generation of macrophages in mice treated with this hormone. Accordingly,* in vitro* production of macrophages from BM progenitors was reduced by calcitonin.

The generally accepted paradigm proposes the recruitment of circulating monocytes to inflammatory site whereupon they differentiate into macrophages (as reviewed in [24, 55, 56]). However, recent studies have reported local proliferation of resident macrophages in response to inflammation [57, 58] and this process seems driven by M-CSF [59]. Calcitonin did not alter M-CSF levels in infected mice; we hypothesize that it may interfere with M-CSF-dependent signalling in certain bone marrow precursor cells and that impaired recruitment of HSPC to the spleen may further compromise myelopoiesis.

It is generally accepted that osteoclasts are the major cells responding to calcitonin (as reviewed in [60]). The hormone inhibits contraction and motility, as well as secretion of acid phosphatase [61–63]. Expression of the calcitonin receptor occurs during the differentiation of myeloid precursors into osteoclasts, requiring simultaneous action of M-CSF and RANKL [64, 65]. As such, the inhibition caused by calcitonin on macrophage differentiation is puzzling and suggests that common BM precursors committing to the monocyte-macrophage differentiation may respond to this hormone.

In summary, based on our preliminary study [33] and our new data, we conclude that the inflammatory response and anemia caused by blood stage infection with* P. c. adami* parasites do not stimulate the activity of osteoclasts. Inhibition of the basal activity of osteoclasts may be sufficient to partially block egression of HSPC from the BM, but this egression seems not essential for the stress erythropoiesis in conditions of acute anemia. The intriguing effects of calcitonin on the differentiation and proliferation of macrophages remain to be characterized.

## Supplementary Material

Figure 1: Represents P.c. adami control and calcitonin-treated mice splenic indexes.
Figure 2: Represents lymphoid and myeloid splenic cell populations at peak infection.
Figure 3: Represents M-CSF concentrations in serum 5, 8 and 10 days post-infection.
Figure 4: Represents the strategy used to gate DiD+ liposome loaded and DID- macrophages (F4-80+ cells).
