# Supplementary material for: Osteoclasts Are Required for Hematopoietic Stem and Progenitor Cell Mobilization but Not for Stress Erythropoiesis in Plasmodium chabaudi adami Murine Malaria
Source: Mediators Inflamm. 2016 Jan 21;2016:3909614. doi: 10.1155/2016/3909614 (PMC4745282; doi:10.1155/2016/3909614)
Supplement: Supplementary file 1 — Figure 1: Represents P.c. adami control and calcitonin-treated mice splenic indexes. Figure 2: Represents lymphoid and myeloid splenic cell populations at peak infection. Figure 3: Represents M-CSF concentrations in serum 5, 8 and 10 days post-infection. Figure 4: Represents the strategy used to gate DiD+ liposome loaded and DID- macrophages (F4-80+ cells). [file 3909614.f1.pdf]

Supplementary figures.

Supplementary figure 1.

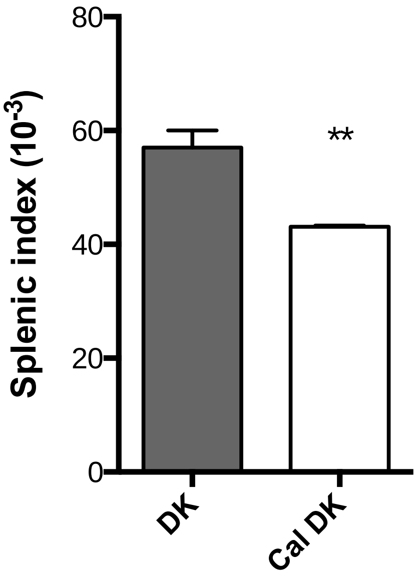

Supplementary figure 1. Effect of calcitonin treatment on splenic index during *Plasmodium* infection. Mice were treated with calcitonin (white bar; n=4) or with PBS (grey bar; n=4) during 5 consecutive days and were infected with *P. c. adami DK parasites* (10^5 pRBCs) on the 3rd day of treatment. Mice were sacrificed at 8 day post-infection and the splenic index was calculated by dividing spleen weight by body weight. Data are mean ± SEM and values are compared using a non-parametric Student *t* test. ***p*<0.01.

Supplementary figure 2.

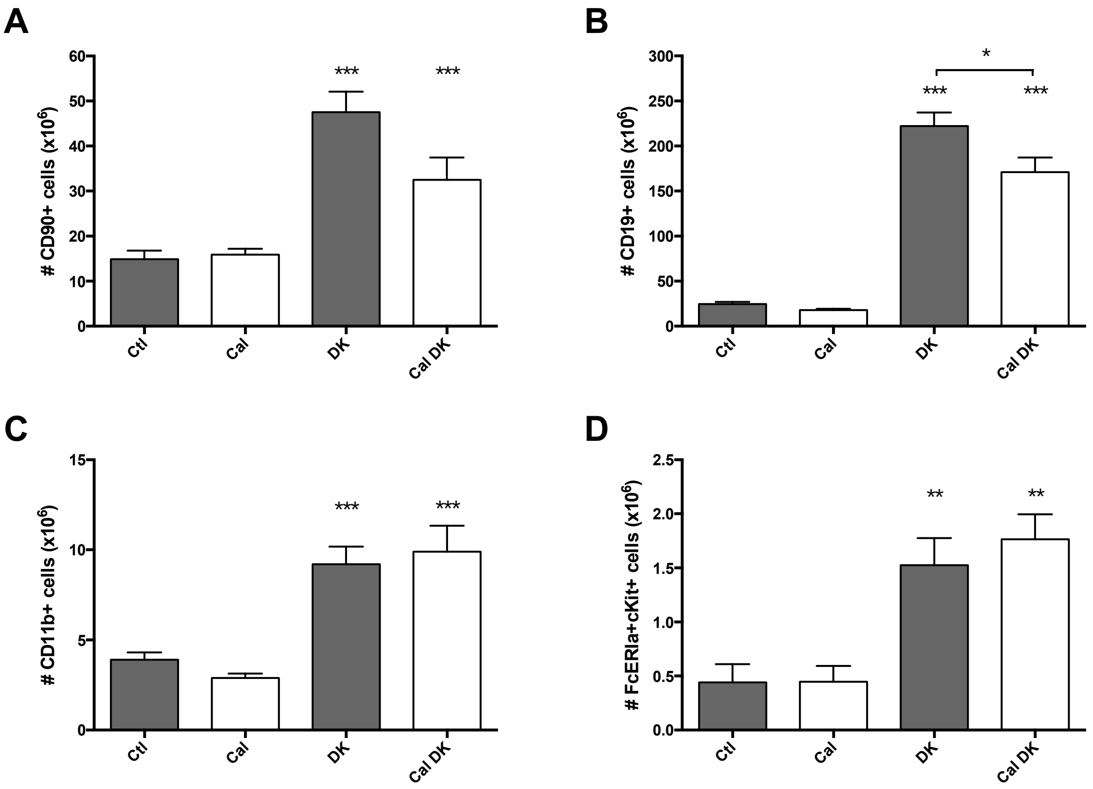

Supplementary figure 2. Effect of calcitonin treatment on lymphoid and myeloid populations in spleen during *Plasmodium* infection. Mice were treated with calcitonin (white bar) or with PBS (grey bar) during 5 consecutive days and infected with *P. c. adami DK parasites* (10^5 pRBCs) on the 3rd day of treatment (7-11 mice per group). Mice were sacrificed at 8 day post infection and 3 uninfected controls were included; the spleen was aseptically recovered for analysis of number of $CD90^+$ (A) and $CD19^+$ cells (B) within the lymphocyte population. The numbers of $CD11b^+$ cells (C) and $FcERIa^+cKit^+$ cells (D) were also assessed in spleen. Data are mean ± SEM and values are compared to respective uninfected control mice and between the two infected groups using a one-way ANOVA test. $*p<0.05$; $**p<0.01$; $***P<0.001$.

Supplementary figure 3.

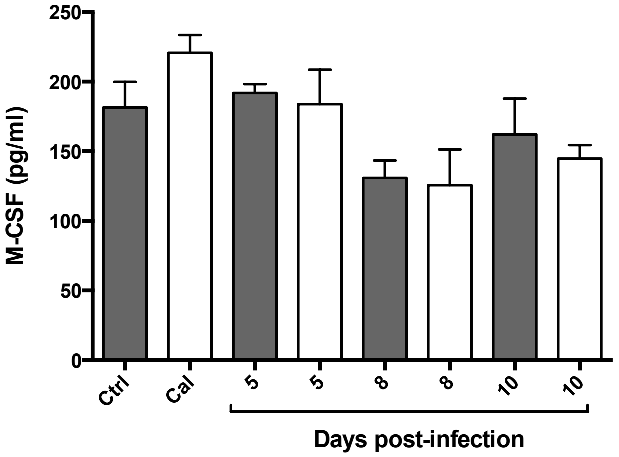

Supplementary figure 3. Effect of calcitonin on plasma level of Macrophage–Colony Stimulating Factor (M-CSF) during *Plasmodium* infection. Mice were treated with calcitonin (white bars; n=4) or with PBS (grey bars; n=4) during 5 consecutive days and were infected with *P. c. adami DK parasites* (10^5 parasitized RBCs) on the 3rd day of treatment. Mice were sacrificed at 5, 8 and 10 days post infection and M-CSF was assessed in plasma by ELISA. Data are mean ± SEM and values are compared to respective controls using a one-way ANOVA test.

Supplementary figure 4.

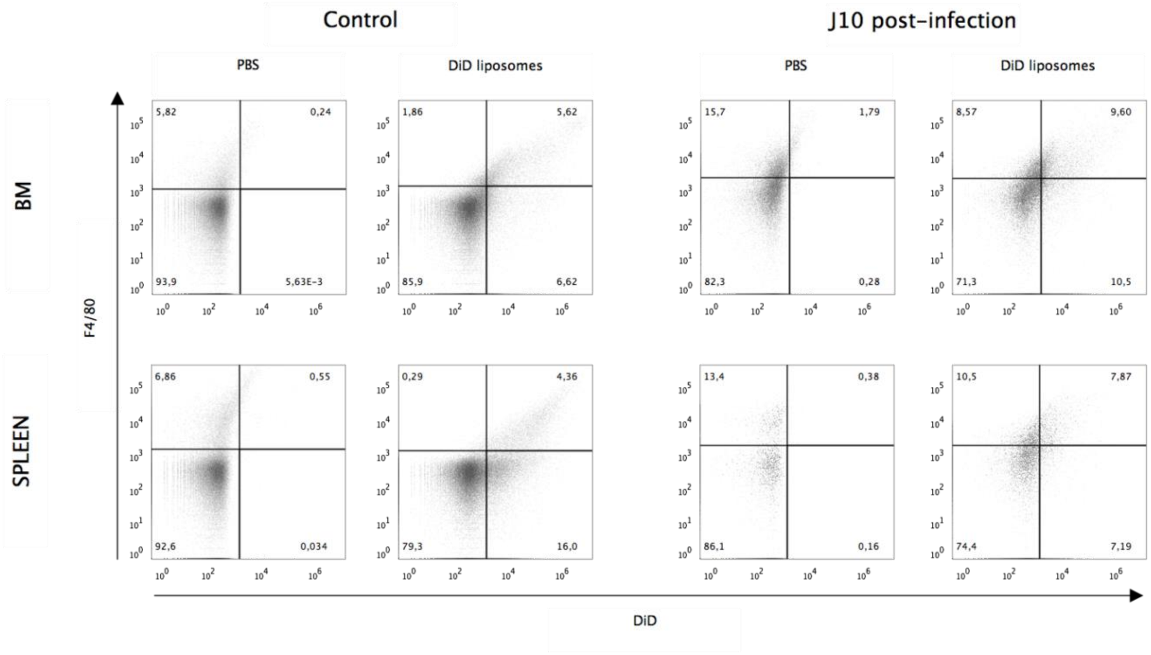

Supplementary figure 4. Identification panel of mature phagocytic macrophages in spleen and BM. Three days prior to infection, mice were intravenously injected with DiD-loaded liposomes or PBS-loaded liposomes as control. At 10 day post infection, infected and control mice were sacrificed and spleen and BM were aseptically recovered for analysis of mature macrophages (F4/80$^+$DiD$^+$) or *de novo* macrophages (F4/80$^+$DiD$^-$) by flow cytometry.
